# Polymerization of Hexene-1 and Propylene over Supported Titanium–Magnesium Catalyst: Comparative Data on the Polymerization Kinetics and Molecular Weight Characteristics of Polymers

**DOI:** 10.3390/polym15010087

**Published:** 2022-12-26

**Authors:** Mikhail Matsko, Ludmila Echevskaya, Vladimir Zakharov

**Affiliations:** Boreskov Institute of Catalysis, Pr. Lavrentieva 5, Novosibirsk 630090, Russia

**Keywords:** hexene-1 polymerization, propylene polymerization, titanium-magnesium catalyst, molecular weight, molecular weight distribution polyhexene, polypropylene

## Abstract

Data are presented on the great differences of the kinetics of hexene-1 and propylene polymerization over the same supported titanium–magnesium catalyst, as well as molecular weight and molecular weight distribution of the polymers produced. It is found that the composition of cocatalysts (AlEt_3_ or Al(i-Bu)_3_ greatly affects the kinetics of hexene-1 polymerization and molecular weight distribution of polyhexene, contrary to data obtained at propylene polymerization. The presence of hydrogen at hexene-1 polymerization leads to a much higher increase of activity in comparison with propylene polymerization. Possible reasons for these differences are discussed on the basis of experimental results.

## 1. Introduction

Polyhexene (PH) is a relatively new promising polymer, which may be applied in various fields. The most well-known application is ultrahigh molecular weight polyhexene with the molecular weight higher than 10 × 10^6^ g/mol, which is employed as drag reducing additives to reduce hydrodynamic resistance in oil pipelines [1,2]. At the same time, PH with different molecular weights necessary for other applications can be obtained by varying the polymerization conditions and the composition of catalysts used for PH production.

Catalysts of different compositions can be employed to produce polyhexene; among them are traditional Ziegler-Natta type catalysts, supported titanium-magnesium catalysts (TMC) [3,4,5,6,7,8,9,10,11,12,13,14,15,16], and homogeneous metallocomplex catalysts [17,18,19,20]. Kinetics of hexene-1 polymerization over Ziegler-Natta catalysts with different composition and data on the effect of catalysts’ composition, and polymerization conditions on the molecular weight and molecular weight distribution of polyhexene are presented and discussed in refs. [3,4,5,6,7,8,9,10,11,12,13,14,15,16,17,18,19,20]. Modern TMC, which are commonly used for stereospecific propylene polymerization, are highly active in hexene-1 polymerization also. It was shown in ref. [16] that variation of the composition of a catalytic system and polymerization conditions makes it possible to obtain polyhexene with the molecular weight from 7 × 10^4^ to 2 × 10^6^ g/mol, controllable molecular weight distribution (MWD) (M_w_/M_n_ in the region from 3.7 to 25), and different isotacticity (the content of mmmm pentads from 56 to 96%).

The analysis of literature data shows that polymerization of hexene-1 over titanium-magnesium catalysts strongly differs from the results obtained for propylene. In particular, propylene polymerization over the TMC containing dibutyl phthalate as a stereoregulating component and triethylaluminum (AlEt_3_) as a cocatalyst leads to the formation of polypropylene (PP) with quite narrow MWD (M_w_/M_n_ = 4–6). However, polymerization of hexene-1 over a similar TMC with the AlEt_3_ cocatalyst yields polyhexene with a broad MWD (M_w_/M_n_ = 15–25) [12]. When the AlEt_3_ cocatalyst is replaced by tri-isobutylaluminium (Al(i-Bu)_3_), polyhexene with a narrower MWD (M_w_/M_n_ = 3.7–15) is formed over the same TMC. It should be noted that data concerning the effect of the cocatalyst composition (AlEt_3_ or Al(i-Bu)_3_) on the polymerization kinetics of propylene and molecular weight characteristics of polypropylene are absent in the literature. We think that possible reasons of differences in the polymerization kinetics of hexene-1 and propylene and in the molecular weight characteristics of the produced polymers could be revealed by a more detailed investigation of the polymerization kinetics of these monomers and molecular weight characteristics of polyhexene and polypropylene obtained on similar samples of titanium-magnesium catalyst.

In this paper, we have presented comparative data on the polymerization kinetics of hexene-1 and propylene over the same supported TMC used for stereospecific polymerization of propylene, as well as the data on molecular weight and MWD of polyhexene and polypropylene obtained under variation of polymerization conditions and cocatalyst composition. The data were used to discuss possible reasons for the great differences in the polymerization kinetics of these monomers and in the MWD of PH and PP samples.

## 2. Materials and Methods

The stereospecific titanium–magnesium catalyst used in this study contains TiCl_4_ supported onto MgCl_2_ (2.4 wt% of Ti) and dibutyl phthalate (DBP, 12 wt%) as the internal donor; average particle size of catalyst is 20 µm.

1-Hexene polymerization was carried out in a 1 L steel reactor in heptane. Catalyst concentration was 0.04 g L^−1^; tri-ethylaluminum (AlEt_3_) or tri-isobutylaluminum (Al(i-Bu)_3_) were used as cocatalysts. Cocatalyst concentration was 5–6 mmol L^−1^. Propyl-tri-methoxysilane (PTMS) was used at polymerization as an external donor with molar ratio AlR_3_/PTMS equal to 10–12. Additional data on polymerization conditions and MWD of polyhexene are presented in Tables. Activity of TMC was calculated according to the yield of the polymer, with allowance of monomer conversion during polymerization.

Propylene polymerization was performed in the 1L steel reactor in heptane at constant propylene pressure (6 bar) and hydrogen pressure (0.14 bar). AlEt_3_ and Al(i-Bu)_3_ was used as a cocatalyst ([AlR_3_] = 4 mmol L^−1^); the catalyst concentration was 0.032 g L^−1^. Cyclohexylmethyl-di-methoxysilane (CHMDMS) was used as an external donor.

The method of MWD determination is described in [16].

## 3. Results and Discussion

**Figure 1:**
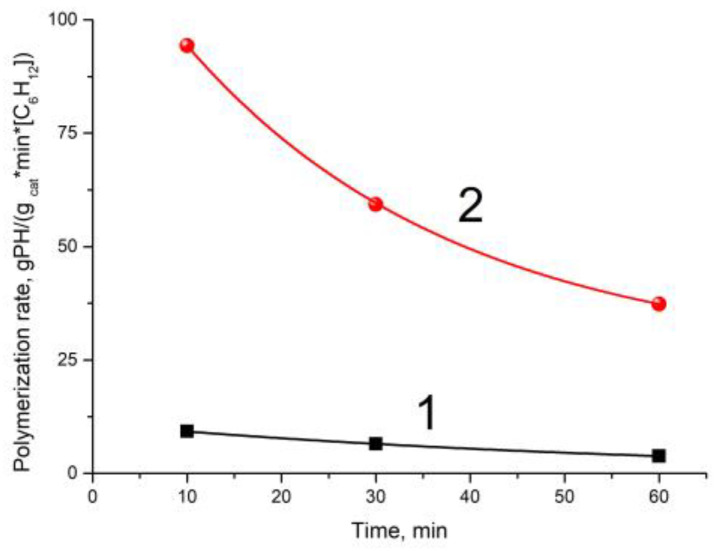
Dependence of hexene-1 polymerization rate on the polymerization duration with cocatalyst AlEt_3_ at polymerization in the absence of hydrogen (curve 1) and in the presence of H_2_ (curve 2) (see polymerization conditions in Table 1).

Our earlier study [15] revealed that in the case of hexene-1 polymerization over titanium–magnesium catalyst, the composition of cocatalyst (AlEt_3_ or Al(i-Bu)_3_) substantially affects the MWD of the produced polyhexene. In particular, the use of AlEt_3_ as a cocatalyst leads to the formation of a polyhexene with a broader molecular weight distribution in comparison with MWD of polyhexene obtained in the presence of the Al(i-Bu)_3_ cocatalyst. More detailed data on MWD of polyhexene produced with the AlEt_3_ cocatalyst under variation of polymerization time and polyhexene yield in the experiments performed in the absence or presence of hydrogen are listed in Table 1. According to these data, dependences of the polymerization rate vs. polymerization duration were obtained for the experiments carried out in the presence or absence of H_2_ (Figure 1).

It is seen that MWD of polyhexene narrows with an increase in polymerization time (polymer yield); however, the MWD remains quite broad (M_w_/M_n_ = 15 and 10 in Exps. 3 and 6, Table 1) even at high yields of the polymer. The introduction of hydrogen into polymerization leads to the decrease of the molecular weight and significantly narrows the MWD for both cocatalysts (Table 1 and Table 3).

It is seen that the rate of hexene-1 polymerization with the AlEt_3_ is higher during the initial period of polymerization (10 min) and then decreases with time (Table 1 and Figure 1). As was noted earlier [15], hydrogen introduction into polymerization of hexene-1 leads to a sharp increase in the polymerization rate (ca. 10-fold, Table 1).

**Figure 2:**
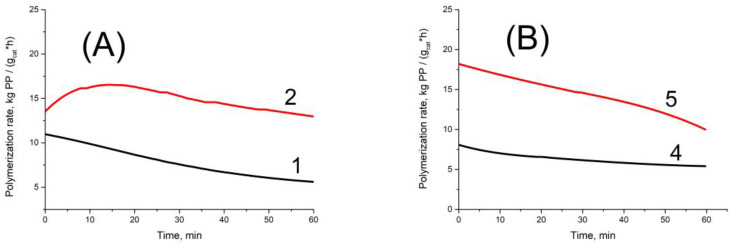
Kinetic curves of propylene polymerization over TMC with AlEt_3_ (**A**) and Al(i-Bu)3 (**B**) as cocatalysts at polymerization in the absence of hydrogen (curves 1 and 4) and in the presence of hydrogen (curves 2 and 5). Numbers of curves correspond to numbers in Table 2.

Data on the polymerization rates of propylene and hexene-1 in the case of the same titanium–magnesium catalyst and different cocatalysts (AlEt_3_ and Al(i-Bu)_3_), as well as the data on MWD and isotacticity of the produced polymers are listed in Table 2 and Table 3. Figure 2 displays typical kinetics curves of propylene polymerization (time dependences of polymerization rate) that were obtained with AlEt_3_ and Al(i-Bu)_3_ cocatalysts in the presence or absence of H_2_. A comparison of the results presented in Table 2 and Table 3 reveals the following peculiarities of polymerization of these monomers over titanium–magnesium catalysts.

In polymerization of propylene, the activity of TMC (polymerization rate) is close for AlEt_3_ and Al(i-Bu)_3_ cocatalysts at polymerization in the absence or presence of H_2_ (compare Exps. 1 and 2, 4 and 5 in Table 2). At the same time, in polymerization of hexene-1 with the AlEt_3_ as a cocatalyst, the catalyst activity is much lower compared to the Al(i-Bu)_3_ cocatalyst, especially at polymerization in the absence of H_2_ (Exps. 1 and 3 in Table 3). However, in the presence of H_2_ the catalyst activity at polymerization of hexene-1 sharply increases by a factor of 12–32 (Table 3) and approaches the activity of the same catalyst in polymerization of propylene (compare Exps. 2, 3, 5, 6 in Table 2 with Exps. 2 and 4 in Table 3). In the case of propene polymerization, activity of the catalyst increases upon hydrogen introduction only by a factor of 1.5–2. Thus, the effect of the catalyst activity growth due to hydrogen introduction at polymerization of hexene-1 is much more pronounced than in the case of propylene polymerization. In refs. [21] it was shown that the increase of TMC activity during propene polymerization in the presence of H_2_ is associated with reactivation of temporarily inactive, so called “dormant” sites, which are formed due to 2,1-addition of propylene to the propagating polymer chain. Our data on a more abrupt increase of the TMC activity after hydrogen introduction at polymerization of hexene-1 testify that the fraction of dormant sites formed during polymerization of hexene-1 in the absence of hydrogen is much greater as compared to polymerization of propylene. In this case, the reactivity of propylene and hexene-1 in the polymer chain propagation reaction should be estimated from the data on the catalyst activity obtained at polymerization in the presence of H_2_ (in the absence of dormant sites). Data obtained under the indicated conditions using the Al(i-Bu)_3_ cocatalyst (Exps. 5, 6 in Table 2 and Exp. 4 in Table 3) demonstrate close reactivities of these monomers in the polymer chain propagation reaction.

In the case of propylene polymerization, the cocatalyst type affects isotacticity of polypropylene; the application of Al(i-Bu)_3_ as a cocatalyst significantly decreases isotacticity of polypropylene (Table 2, Exps. 2 and 5). At the same time, at polymerization of hexene-1, isotacticity of polyhexene-1 does not depend on the composition of cocatalyst (Table 3, Exps. 2 and 4).

Polypropylene samples produced in the presence of H_2_ with AlEt_3_ or Al(i-Bu)_3_ cocatalysts have close molecular weights (Table 2, Exps. 2 and 5). These polymers also have close polydispersity values (Mw/Mn = 4.0–5.4). Data on the MWD of polyhexene (Table 3) greatly differ from the data obtained for polypropylene. Polyhexene produced in the absence of hydrogen with the AlEt_3_ cocatalyst has a much lower molecular weight and a very broad MWD (M_w_/M_n_ = 17.4) compared to PH produced with the Al(i-Bu)_3_ cocatalyst (Table 3, Exps. 1 and 3). The introduction of H_2_ leads to a sharp decrease in the molecular weight of polyhexene and to some narrowing of MWD in the case of AlEt_3_ cocatalyst; nevertheless, the M_w_/M_n_ values for PH produced with the AlEt_3_ cocatalyst remain much higher than the values for PH produced with Al(i-Bu)_3_ cocatalyst (Table 3, compare Exps. 1 and 2 with Exps. 3 and 4).

Reactivities of propylene and hexene-1 in the chain transfer reactions can be compared using the polymerization degree (P_n_) data for polypropylene and polyhexene obtained under close conditions in the presence of hydrogen. In the case of propylene and hexene polymerization with the AlEt_3_ cocatalyst at a low hydrogen content, the polymerization degree of PH is much lower than that of PP (200 and 1900, respectively) (Exp. 2 in Table 2 and Exp. 2 in Table 3). However, in polymerization with the Al(i-Bu)_3_ cocatalyst, the polymerization degree of PP and PH are close (500 and 440) (Exp. 6 in Table 2 and Exp. 4 in Table 3). Presumably, these results may be caused by a great contribution of the chain transfer reaction, with AlEt_3_ at a low hydrogen content to polymerization degree of polyhexene in comparison with propylene polymerization. At the same time, during polymerization of propylene and hexene with the Al(i-Bu)_3_ cocatalyst at an increased hydrogen content, the contribution of the chain transfer reaction with cocatalyst to the polymerization degree is insignificant. For the indicated polymerization conditions, polymerization degree is determined by the rate constant ratio for chain propagation and polymer chain transfer with H_2_. Data on the close polymerization degree of PP and PH obtained for such polymerization conditions indicate that the ratios of these rate constants for polymerization of propylene and hexene-1 over TMC are also close.

Data presented in Table 2 and Table 3 show the essential differences in the MWD of polypropylene and polyhexene are observed only at polymerization with cocatalyst AlEt_3_. This cocatalyst is the efficient agent of the chain transfer reaction in the case of ethylene, propylene, and hexene-1 polymerization in the absence of hydrogen. In case of ethylene polymerization over TMC it was proposed in [22] that AlEt_3_ is able to form temporarily inactive (“dormant”) sites due to the reversible adsorption on active sites (AS). This reaction proceeds additionally to the decrease of molecular weight of polyhexene and broadening of MWD [22]. Probably the contribution of this reaction increases at polymerization of hexene-1 compared to ethylene and propylene polymerization due to the elevated concentration of AlEt_3_ on the catalyst surface. This phenomenon may be caused by the formation of a homogeneous reaction medium (a polyhexene solution in heptane) in distinction to a heterogeneous medium that appears when solid polypropylene particles are formed as a suspension in a heptane medium.

Earlier in our paper [15], we have presented data concerning the effect of reaction temperature on the polymerization rate of hexene-1 over TMC with cocatalysts AlEt_3_ and Al(i-Bu)_3_ in the presence or absence of H_2_ at polymerization. We have found in most cases that the polymerization rate decreases when the reaction temperature is increased from 30 °C up to 70 °C. Due to this unusual effect of decreasing polymerization rate with elevation of the reaction temperature, the calculated effective activation energies for polymerization rate (E_eff_) have anomalous negative values. The most pronounced effect on the temperature dependence of hexene-1 polymerization rate and the calculated values of E_eff_ was exerted by the composition of cocatalyst and the presence of H_2_ during polymerization (Table 4).

Data concerning the effect of reaction temperature on the polymerization rate of hexene-1 greatly differ from the results obtained for propylene polymerization over the same titanium–magnesium catalyst with AlEt_3_ as a cocatalyst (Table 5).

One can see that in the case of propylene, the polymerization rate substantially increases with elevation of the reaction temperature. The E_eff_ values (32–45 kJ/mol) calculated from these data are in the region known for polymerization of propylene over TMC. The indicated values strongly differ from the anomalous negative values of E_eff_ (−2.2 and −21 kJ/mol) calculated for polymerization of hexene-1 with AlEt_3_ or Al(i-Bu)_3_ cocatalyst in the absence of H_2_ (Table 4, Exps. 1–3 and Exps. 4–6). The “normal” positive value of E_eff_ (20 kJ/mol, Exps. 7–8 in Table 4) was obtained only for polymerization with the Al(i-Bu)_3_ cocatalyst in the presence of H_2_.

The data on the decreasing of hexene-1 polymerization rate with an increase of polymerization temperature (Table 4, Exps. 1–3 and 4 and 6) may be related to a decrease in the number of AS that occurs at elevation of polymerization temperature under the indicated conditions. The appearance of this effect is determined by the composition of cocatalyst (AlEt_3_) and the absence of hydrogen at polymerization.

We suppose that the decrease of the number of AS and, correspondingly, the decrease of polymerization rate of hexene-1 with elevation of the reaction temperature at polymerization with AlEt_3_ cocatalyst may be associated with the state of reaction medium. In this case, polymerization proceeds in a homogeneous medium with the formation of a polyhexene solution in heptane. At such state of the reaction medium, the concentration of AlEt_3_ on the catalyst surface corresponds to its concentration in the polyhexene solution, in distinction to propylene polymerization, when a layer of semicrystalline polymer is formed on the catalyst surface and the concentration of AlEt_3_ on the catalyst surface is much lower than its concentration in the heptane solution. The high AlEt_3_ concentration on the catalyst surface at polymerization of hexene-1 may decrease the number of AS at elevation of polymerization temperature due to the reduction of a part of Ti^3+^ ions in active sites to the inactive Ti^2+^ sites.

The revealed substantial effect of hydrogen on the dependence of hexene-1 polymerization rate of the reaction temperature (Table 4, Exps. 4–6 and 7–9) may be related to the known phenomenon consisting in the formation of “dormant” sites at polymerization of α-olefins over TMC in the absence of H_2_ and the possibility of their reactivation in the presence of H_2_ [21]. The dormant sites are formed at polymerization of α-olefins in the absence of hydrogen as a result of 2,1-addition of α-olefin to the propagating polymer chain. These sites are reactivated upon interaction with hydrogen, thus enhancing the activity at polymerization of propylene and hexene-1 in the presence of H_2_. As was noted above, in the case of hexene-1 polymerization, the fraction of dormant sites formed in the absence of hydrogen and, accordingly, the enhancement of activity after hydrogen introduction are much higher than in the case of propylene polymerization. Presumably, the fraction of dormant sites formed in the absence of hydrogen depends on the polymerization temperature and increases with its elevation. This occurs because the reaction of α-olefin 2,1-addition to the propagating chain has a higher activation energy compared to the normal 1,2-addition; so, the fraction of dormant sites in the absence of H_2_ increases with the elevation of polymerization temperature. The effect of hydrogen on the catalyst activity at different temperatures of hexene-1 polymerization and, accordingly, on the estimated E_eff_ values manifests itself most clearly in experiments with the Al(i-Bu)_3_ cocatalyst (Table 4). It is seen that at polymerization in the absence of hydrogen (Exps. 4–6) the activity weakly decreases when the polymerization temperature is increased from 30 °C to 70 °C (E_eff_ = −2.2 kJ/mol). At the same time, during polymerization in the presence of hydrogen (Exps. 7–9, Table 4) the activity noticeably increases with the elevation of polymerization temperature from 30 °C to 70 °C (E_eff_ = 20 kJ/mol).

Thus, at polymerization of hexene-1 over TMC the composition of cocatalyst and the presence of H_2_ strongly affect the dependence of polymerization rate on the reaction temperature and determine the possibility of a substantial decrease in the number of active sites with an increasing polymerization temperature from 30 °C up to 70 °C. This fact leads, firstly, to the appearance of anomalous negative values of the apparent activation energy of polymerization and, secondly, to a pronounced difference in the calculated E_eff_ values for different compositions of the catalytic system and reaction medium. In particular, according to the data of Table 4, the calculated values of E_eff_ vary from −21 to 20 kJ/mol.

## 4. Conclusions

Data are obtained on the great differences of the kinetics of hexene-1 and propylene polymerization over the TMC as well as the molecular weight and molecular weight distribution of polymers produced. It is found that the composition of cocatalysts (AlEt_3_ or Al(i-Bu)_3_) greatly affects the molecular weight and MWD of polyhexene, contrary to polypropylene. Polyhexene produced with AlEt_3_ cocatalyst has a lower molecular weight and broader MWD (M_w_/M_n_ = 10–22) in comparison with polyhexene produced with Al(i-Bu)_3_ cocatalyst (Mw/M_n_ = 4–5). Polypropylene produced with both AlEt_3_ and Al(i-Bu)_3_ cocatalysts has a similar MWD (M_w_/M_n_ = 4–5.5). In the case of propylene polymerization, the activity of TMC is similar with AlEt_3_ and Al(i-Bu)_3_ cocatalysts, but in the case of hexene-1, the polymerization activity is much higher with Al(i-Bu)_3_ cocatalyst in comparison with AlEt_3_ cocatalyst.

The addition of hydrogen at hexene-1 polymerization leads to the great increase of activity (10–32 times), but in the case of propylene polymerization, activity increases only 1.5–2 times. These results show that the fraction of “dormant” sites formed at hexene-1 polymerization in the absence of H_2_ is much higher in comparison with propylene polymerization. Note that the activity of TMC with Al(i-Bu)_3_ cocatalyst in the presence of H_2_ is close to the activity of this catalyst at propylene polymerization in the presence of H_2_.

In the case of hexene-1 polymerization, we have found the unusual effect of the decrease of polymerization rate at increase of polymerization temperature from 30 °C up to 70 °C. Due to this effect, the activation energies calculated for polymerization rate (E_eff_) have anomalous negative values within the range from −2.2 kJ/mol to −21 kJ/mol. These values depend on the composition of the cocatalyst and the presence of H_2_; the maximal negative value (−21 kJ/mol) is observed for polymerization with AlEt_3_ cocatalyst in the absence of hydrogen. Note that in the case of propylene polymerization with TEA cocatalyst we have found the usual E_eff_ values (32–45 kJ/mol). So, two main factors—the composition of the cocatalyst and the presence of H_2_ leads to differences in the kinetics of hexene-1 and propylene polymerization and molecular mass characteristics of polymers. 

The strong effect of cocatalyst AlEt_3_ on the activity, molecular weight, and MWD of polyhexene may be caused by the formation of a homogeneous reaction medium (solution of polyhexene in heptane), in distinction of a heterogeneous medium when solid polypropylene particles are formed as a slurry in heptane. In this case, the concentration of AlEt_3_ on the surface of the catalyst is much higher at hexene-1 polymerization in comparison with one at propylene polymerization. High AlEt_3_ concentration on the surface of a catalyst leads to a decrease in the number of active sites (activity of catalyst), especially at high temperatures (70 °C) and an increase in the rate of chain transfer reaction with AlEt_3_.

In the case of hexene-1 polymerization in the absence of H_2_ activity is much lower in comparison with propylene polymerization because a higher fraction of “dormant” sites formed at hexene-1 polymerization in comparison with propylene polymerization. The addition of H_2_ leads to the reactivation of “dormant” sites and an increase of activity in 10–30 times at hexene-1 polymerization. In this case, activity of TMC with Al(i-Bu)_3_ cocatalyst in the presence of H_2_ is close at hexene-1 and propylene polymerization.

## Figures and Tables

**Table 1:** Data on the effect of polymerization duration on the polymerization rate and molecular weight of polyhexene upon hexene-1 polymerization with AlEt_3_ as cocatalyst.

Exp. ^1^ No.	Hydrogen Presence	Polymerization Duration, min	G ^3^	R_p_^4^	M_n_, kg/mol	M_w_, kg/mol	M_w_/M_n_
1 2 3	− − −	10 30 60	0.18 0.37 0.43	9.2 6.5 3.8	22 27 32	490 510 490	22 19 15
4 ^2^ 5 ^2^ 6 ^2^	+ + +	10 30 60	1.5 2.4 2.8	94 59 37	17 16 15	230 170 150	13.5 11 10

^1^ Polymerization at 70 °C, [C_6_H_12_] = 2 M. ^2^ H_2_ pressure is 0.1 bar in Exps 4–6. ^3^ Polymer yield, kg PH/g cat. ^4^ g PH/(g cat. × min × mol C_6_H_12_).

**Table 2:** Data on the propylene polymerization over TMC with AlEt_3_ or Al(i-Bu)_3_ cocatalysts.

Exp. No.	Cocatalyst	P_H2_, Bar	G ^1^	R_p_^2^	II ^3^	M_n_, kg/mol	P_n_^4^	M_w_, kg/mol	M_w_/M_n_
1 2 3	AlEt_3_	─ 0.16 0.5	7.8 14.5 13.7	3100 5800 5400	─ 97 ─	─ 81 35	─ 1900 800	─ 320 140	─ 4.0 4.0
4 5 6	Al(i-Bu)_3_	─ 0.16 0.5	6.3 14.4 11.1	2600 5700 4300	─ 88 ─	─ 75 20	─ 1800 500	─ 300 110	─ 4.0 5.4

Polymerization at 70 °C, [C_3_H_6_]—2 mol/L, [AlR_3_]—4 mol/L, AlR_3_/ED = 20, polymerization duration—1 h. ^1^ Polymer yield, kg PP/g cat. ^2^ Polymerization rate, mol C_3_H_6_/(mol Ti × min × mol C_3_H_6_). ^3^ Isotacticity of PP, %. ^4^ Polymerization degree.

**Table 3:** Data on the hexene-1 polymerization over TMC with different cocatalysts.

Exp. No.	Cocatalyst	P_H2_, Bar	τ_p_^1^, min	G ^2^	R_p_^3^	II ^4^	M_n_, kg/mol	P_n_^5^	M_w_, kg/mol	M_w_/M_n_
1 2	AlEt_3_	─ 0.2	60 10	0.2 1.2	80 2800	─ 95	20 12	400 200	350 170	17.5 14.0
3 4	Al(i-Bu)_3_	─ 0.5	60 10	1.0 2.0	380 4800	─ 96.5	600 37	7140 440	2500 185	4.2 5.0

Polymerization at 70 °C, [C_6_H_12_]—1 mol/L, [AlR_3_]—6 mmol/L, AlR_3_/ED = 10. ^1^ Polymerization duration, min. ^2^ PH yield, kg/g cat. ^3^ Polymerization rate, mol C_6_H_12_/(mol Ti × min × mol C_6_H_12_). ^4^ Content of [mmmm] pentad. ^5^ Degree of polymerization.

**Table 4:** Data on the influence of reaction temperature on the rate of hexene-1 polymerization and values of effective activation energy (E_eff_) upon polymerization over TMC with different cocatalysts in the presence and absence of hydrogen.

Cocatalyst	Exp. No.	T, °C	H_2_	R_p_^1^	E_eff_^2^, kJ/mol
AlEt_3_	1 2 3	30 50 70	- - -	9.6 7.6 3.3	−21
Al(i-Bu)_3_	4 5 6	30 50 70	- - -	66 78 59	−2.2
Al(i-Bu)_3_	7 8 9	30 50 70	+ + +	120 230 300	20

Polymerization at [C_6_H_12_]—1 mol/L, polymerization duration 60 min in Exps. 1–3 and 30 min in Exps. 4–6 and 10 min in Exps. 7–9. ^1^ g PH/(g cat. × min × mol C_6_H_12_). ^2^ Effective activation energy.

**Table 5:** Data on the influence of reaction temperature on the rate of propylene polymerization and values of effective activation energy (E_eff_) upon propylene polymerization over TMC with AlEt_3_ cocatalyst in the presence and absence of H_2_.

Exp. No.	T, °C	H_2_	R_p_^1^	E_eff_, kJ/mol
1 2 3	30 50 70	+ + +	27 70 220	45.2
4 5 6	30 50 70	- - -	27 63 117	31.7

Polymerization with cocatalyst AlEt_3_, polymerization duration 30 min. ^1^ Polymerization rate, g PP/(g cat. × min × mol C_3_H_6_).

## Data Availability

Data presented in this study are available on request from the corresponding author.
